# Network-Level Brain Dysfunction Beyond the Lesion: A Rare Case of Monakow Syndrome Presenting With Fluctuating Consciousness and Left Hemispatial Neglect Due to Right Anterior Choroidal Artery Infarction

**DOI:** 10.7759/cureus.98185

**Published:** 2025-11-30

**Authors:** Godai Yawata, Asato Tsuji, Mariko Takata, Tetsuya Oda, Hirotoshi Hamaguchi

**Affiliations:** 1 Department of Neurology, Kitaharima Medical Center, Ono, JPN

**Keywords:** anterior choroidal artery, diaschisis, hemispatial neglect, monakow syndrome, network neuroscience

## Abstract

Anterior choroidal artery (AChA) infarction typically manifests as the classical triad of contralateral hemiparesis, hemisensory loss, and homonymous hemianopia (Monakow syndrome). We report a rare case of right AChA infarction presenting with fluctuating consciousness and left hemispatial neglect, accompanied by widespread functional suppression of the right cerebral hemisphere. A 65-year-old right-handed man initially exhibited mild dysarthria and left-sided sensory impairment, with no acute lesions on diffusion-weighted MRI. He subsequently developed impaired consciousness and hemispatial neglect. Follow-up MRI demonstrated acute infarcts in the posterior limb of the internal capsule (PLIC) and lateral geniculate nucleus (LGN). Arterial spin labeling (ASL) and N-isopropyl-p-[123I]iodoamphetamine single-photon emission computed tomography (IMP-SPECT) revealed extensive right-hemispheric hypoperfusion, disproportionate to the structural lesion. Recent evidence indicates that the PLIC and LGN function as major network hubs rather than simple relay pathways. Local injury to these hubs may disrupt global network efficiency, producing functional diaschisis. This case highlights the need to consider network-level dysfunction in AChA infarction.

## Introduction

The anterior choroidal artery (AChA), a branch of the internal carotid artery, supplies several strategic deep brain structures, including the posterior limb of the internal capsule (PLIC), lateral geniculate nucleus (LGN), optic radiation, hippocampus, and cerebral peduncle [1]. AChA infarction is uncommon and typically presents with Monakow’s classical triad [2]. However, in the era of connectomics, subcortical lesions are increasingly understood as network-level disturbances rather than isolated focal injuries [3].

The concept of diaschisis, originally introduced by von Monakow, has been revitalized by modern neuroimaging, demonstrating that disruption of critical hubs or white-matter pathways can lead to distant cortical dysfunction [4,5]. The PLIC and LGN are now recognized as integrative hubs connecting motor, sensory, and attentional networks [6]. Injury to these structures may yield higher-order symptoms such as neglect or altered consciousness. We present a rare case of right AChA infarction with fluctuating consciousness and hemispatial neglect, supported by multimodal imaging evidence of widespread right-hemispheric functional suppression.

## Case presentation

A 65-year-old right-handed man with untreated hypertension, chronic alcohol consumption, and long-term smoking awoke with left lower-limb weakness and difficulty using a tablet device. On admission, he was alert with stable vital signs (BP: 186/108 mmHg). Neurological examination revealed mild dysarthria, left lower-limb ataxia, and left-sided sensory impairment. Laboratory studies demonstrated impaired glucose tolerance and vitamin B12 deficiency.

**Figure 1:**
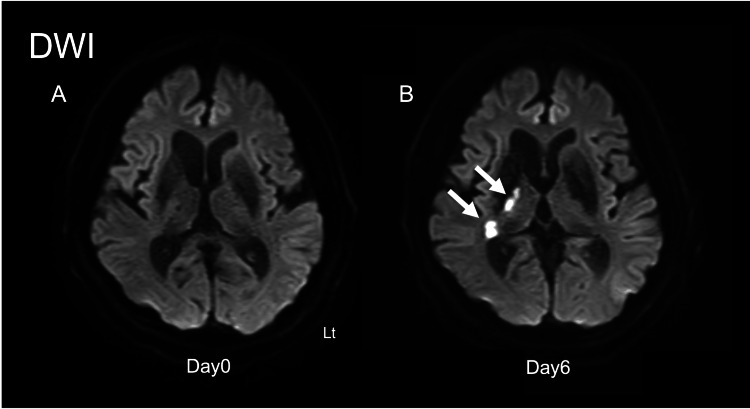
Head MRI images DWI: diffusion-weighted imaging; PLIC: posterior limb of the internal capsule; LGN: lateral geniculate nucleus (A) DWI horizontal section on day 0 of onset. (B) DWI horizontal section on day 6 of onset. The white arrows indicate high signal intensity in the right PLIC and LGN, a finding not observed on day 0

Initial MRI showed no diffusion-restricted lesions, and magnetic resonance angiography (MRA) demonstrated no large-vessel stenosis. Hours after admission, he developed fluctuating consciousness (E4V5M6 → E3V3M6) and left visual inattention. On day 1, MRI revealed acute infarcts in the right PLIC and LGN. These became more prominent by day 6, confirming right AChA infarction (Figure 1).

**Figure 2:**
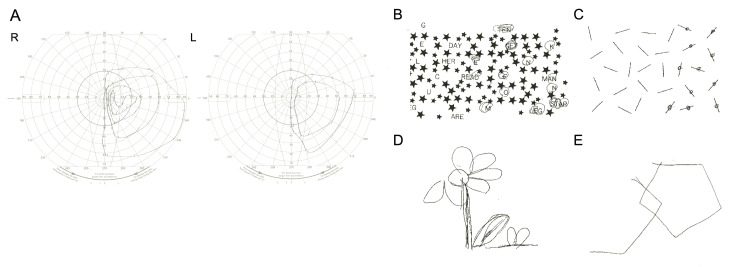
Neuropsychological testing (A) Goldman perimetry shows homonymous hemianopia in the left eye. (B) Alphabet deletion test: Only the right half of the alphabet is processed. (C) Line bisection test: Only the right half of the line is processed, showing a tendency for bisection to be biased to the right. (D) Figure copying: The left half of the flower is missing. (E) Pentagon copying: The left half of the pentagon is missing

No embolic source was identified, and atherothrombotic stroke was suspected. Aspirin (100 mg/day) and argatroban (60 mg/day) were initiated. Goldmann perimetry confirmed left homonymous hemianopia. Neuropsychological testing (star cancellation and line bisection) revealed significant left hemispatial neglect (Figure 2).

**Figure 3:**
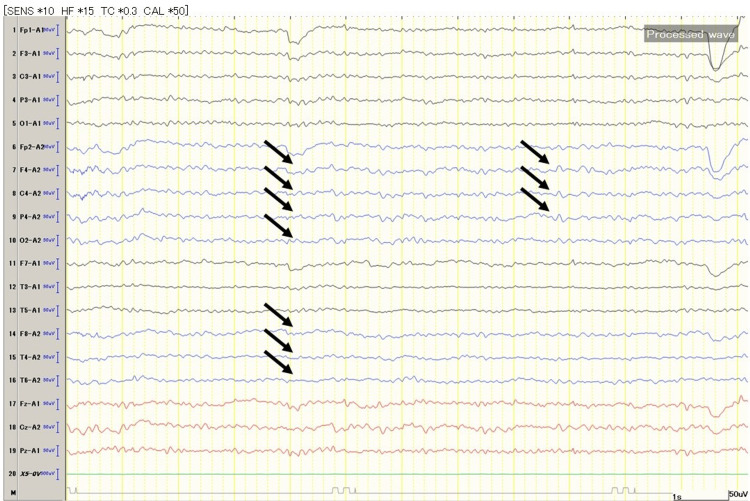
EEG showed slowing over the right hemisphere EEG recorded using the 10-20 system. Recorded using the monopolar recording method. The blue baseline indicates the right hemisphere, the black baseline indicates the right hemisphere. The yellow line represents one second, with a scale bar shown at the bottom right. The black arrows indicate delta wave activity compared to the left hemisphere, suggesting decreased brain function. This activity is occasionally observed in the right hemisphere.

An electroencephalogram (EEG) showed slowing over the right hemisphere without epileptiform discharges, suggesting functional suppression (Figure 3).

**Figure 4:**
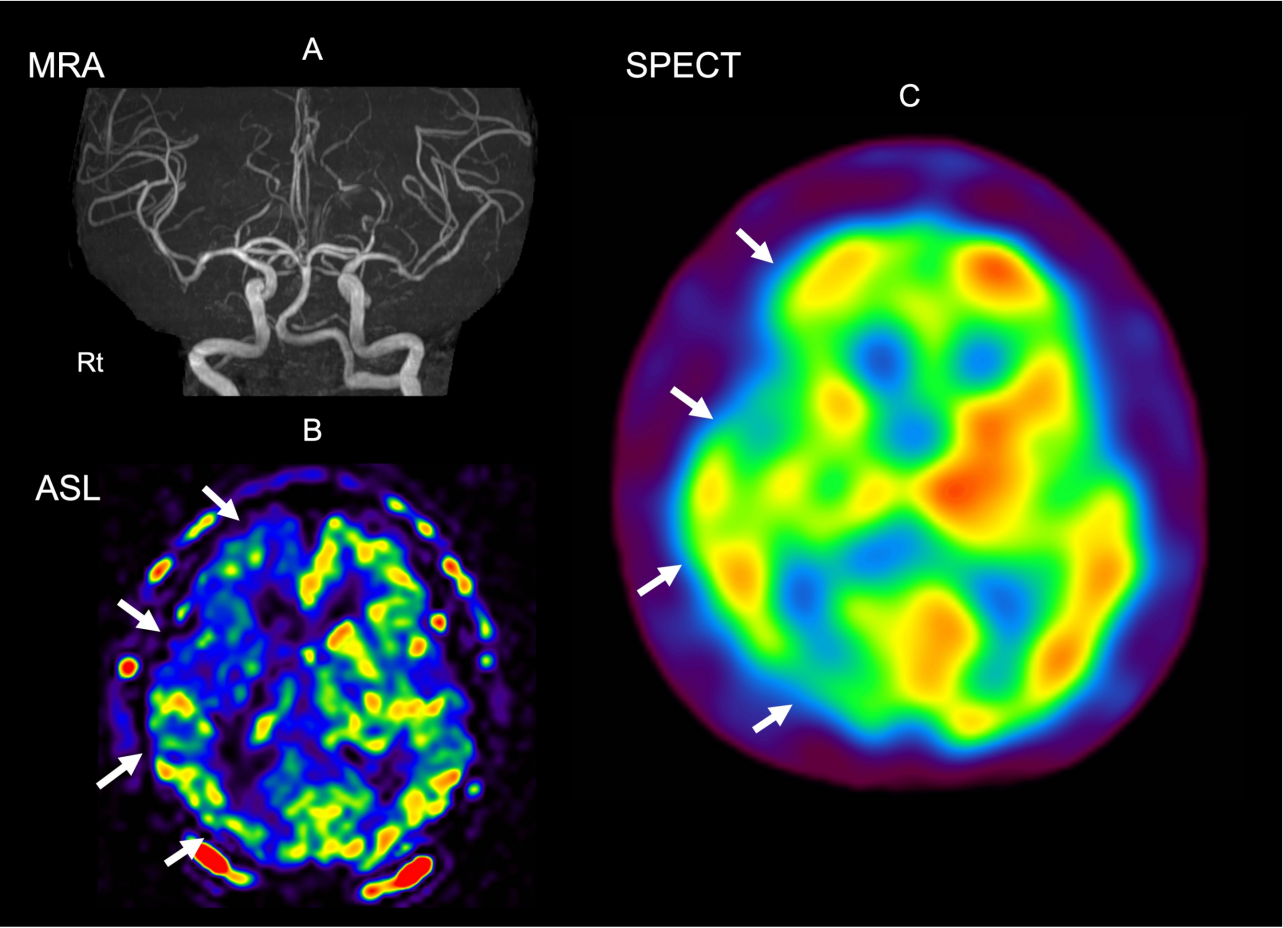
ASL perfusion MRI and IMP-SPECT ASL: arterial spin labeling; MRA: magnetic resonance angiography; IMP-SPECT: N-isopropyl-p-[123I]iodoamphetamine single-photon emission computed tomography (A-B) Day 6 head MRI images. (A) The left image is an MRA image. (B) ASL image. While there is no difference in blood flow, extensive reduced blood flow is observed in the right cerebral hemisphere. The white arrow indicates the area with reduced blood flow. (C) Brain blood flow scintigraphy SPECT (¹²³I-IMP). Reduced blood flow is observed in the transition zone between the right frontal lobe and parietal lobe, and in the right occipital lobe. Red indicates increased blood flow; blue indicates decreased blood flow. The white arrow indicates the area with reduced blood flow

Arterial spin labeling (ASL) perfusion MRI and IMP-SPECT both demonstrated widespread hypoperfusion throughout the right cerebral hemisphere, disproportionate to the small structural infarcts (Figure 4).

The patient was transferred for rehabilitation on day 20 with persistent hemianopia, neglect, mild weakness, and sensory deficits.

## Discussion

Although AChA infarction is traditionally described by Monakow’s triad, contemporary series emphasize a broader clinical spectrum that reflects the strategic location of its perforators, especially toward the PLIC and the LGN. In particular, collateral supply to the PLIC is limited, so even small lesions can produce disproportionate deficits, while involvement of the LGN yields characteristic visual field syndromes [7,8].

From a systems perspective, both the PLIC and LGN act as network “bottlenecks” that integrate and relay high-throughput information between the cortex and subcortex. White-matter connectomics and white-matter functional studies suggest that the internal capsule, particularly the PLIC, participates in hub-like communication supporting motor, sensory, and attentional functions; thus, local damage can reduce global network efficiency out of proportion to lesion size. Similarly, human imaging demonstrates that the LGN is not a passive relay: its activity is modulated by figure-ground context and spatial attention, positioning it within a broader attentional network [9]. These observations provide a mechanistic bridge between a focal AChA lesion and the higher-order deficits (neglect, fluctuation in arousal).

Multimodal perfusion data in our case (ASL and IMP-SPECT) revealed widespread right-hemispheric hypoperfusion far beyond the structural infarct burden, consistent with functional diaschisis [10-12]. Perfusion MRI (ASL) and nuclear imaging can detect diaschisis within hours of stroke onset and track its resolution; importantly, diaschisis correlates with clinical impairments and functional outcomes. Thalamic and cerebellar diaschisis after supratentorial stroke has been characterized using ASL, SPECT, PET, and diffusion imaging, supporting the concept that focal subcortical lesions can depress remote cortical territories through network disconnection.

Hemispatial neglect is commonly attributed to right-hemispheric cortical lesions; however, multiple studies document subcortical neglect after thalamic, striatal, and internal capsule strokes, presumably via disruption of cortico-subcortical attention networks [13-15]. This literature supports the interpretation that our patient’s neglect reflects network-level disconnection rather than a large cortical infarct, with the PLIC and LGN acting as strategic nodes. These observations are consistent with recent network-level models of poststroke diaschisis and reorganization [16,17].

## Conclusions

Right AChA infarction may cause higher-order deficits when small lesions involve strategic subcortical hubs such as the PLIC and LGN. This case demonstrates that even minor focal injury can induce widespread hemispheric suppression consistent with network-level diaschisis. Recognizing this mechanism has direct clinical implications: fluctuations in consciousness or neglect in deep infarcts should not be misinterpreted as infarct extension, seizure activity, or therapeutic failure. Instead, early use of multimodal imaging (ASL, SPECT) and artifact-free EEG can help identify network dysfunction, avoid unnecessary escalation of antiepileptic or antithrombotic therapy, and guide appropriate rehabilitation planning. Therefore, awareness of diaschisis-related physiological depression can meaningfully influence diagnostic accuracy and clinical management.

## References

[1] Tatu L, Moulin T, Vuillier F, Bogousslavsky J (2012). Arterial territories of the human brain. Front Neurol Neurosci.

[2] Helgason C, Caplan LR, Goodwin J, Hedges T 3rd (1986). Anterior choroidal artery-territory infarction. Report of cases and review. Arch Neurol.

[3] Honey CJ, Sporns O, Cammoun L, Gigandet X, Thiran JP, Meuli R, Hagmann P (2009). Predicting human resting-state functional connectivity from structural connectivity. Proc Natl Acad Sci U S A.

[4] Feeney DM, Baron JC (1986). Diaschisis. Stroke.

[5] Carrera E, Tononi G (2014). Diaschisis: past, present, future. Brain.

[6] Thiebaut de Schotten M, Dell'Acqua F, Forkel SJ, Simmons A, Vergani F, Murphy DG, Catani M (2011). A lateralized brain network for visuospatial attention. Nat Neurosci.

[7] Li J, Biswal BB, Wang P, Duan X, Cui Q, Chen H, Liao W (2019). Exploring the functional connectome in white matter. Hum Brain Mapp.

[8] Lee D, Park HJ (2022). A populational connection distribution map for the whole brain white matter reveals ordered cortical wiring in the space of white matter. Neuroimage.

[9] Poltoratski S, Maier A, Newton AT, Tong F (2019). Figure-ground modulation in the human lateral geniculate nucleus is distinguishable from top-down attention. Curr Biol.

[10] Kang KM, Sohn CH, Choi SH (2017). Detection of crossed cerebellar diaschisis in hyperacute ischemic stroke using arterial spin-labeled MR imaging. PLoS One.

[11] Wang J, Pan LJ, Zhou B (2020). Crossed cerebellar diaschisis after stroke detected noninvasively by arterial spin-labeling MR imaging. BMC Neurosci.

[12] Xia C, Zhou J, Lu C, Wang Y, Tang T, Cai Y, Ju S (2021). Characterizing diaschisis-related thalamic perfusion and diffusion after middle cerebral artery infarction. Stroke.

[13] Karnath HO, Himmelbach M, Rorden C (2002). The subcortical anatomy of human spatial neglect: putamen, caudate nucleus and pulvinar. Brain.

[14] Healton EB, Navarro C, Bressman S, Brust JC (1982). Subcortical neglect. Neurology.

[15] Rode G, Pagliari C, Huchon L, Rossetti Y, Pisella L (2017). Semiology of neglect: an update. Ann Phys Rehabil Med.

[16] Grefkes C, Fink GR (2011). Reorganization of cerebral networks after stroke: new insights from neuroimaging with connectivity approaches. Brain.

[17] Siegel JS, Seitzman BA, Ramsey LE (2018). Re-emergence of modular brain networks in stroke recovery. Cortex.

